# Why does mode of conception affect early breastfeeding outcomes? A retrospective cohort study

**DOI:** 10.1371/journal.pone.0265776

**Published:** 2022-03-18

**Authors:** Shiue-Shan Weng, Li-Yin Chien, Yi-Ting Huang, Yen-Tsung Huang, Min Chang

**Affiliations:** 1 Institute of Public Health, National Yang Ming Chiao Tung University, Taipei, Taiwan; 2 Department of Nursing, Collage of Nursing, National Yang Ming Chiao Tung University, Taipei, Taiwan; 3 Institute of Community Health Care, Collage of Nursing, National Yang Ming Chiao Tung University, Taipei, Taiwan; 4 Institute of Statistical Science, Academia Sinica, Taipei, Taiwan; 5 Department of Nursing, Shin Kong Wu Ho-Su Memorial Hospital, Taipei, Taiwan; Texas A&M University College Station, UNITED STATES

## Abstract

**Introduction:**

It is uncertain whether Assisted Reproductive Technology (ART) is associated with an increased risk of poor breastfeeding outcomes and what could be possible mechanisms. This study aimed to examine the effect of mode of conception on breastfeeding outcomes during the first two months postpartum and identify the potential mediating pathways for this relationship.

**Methods:**

A retrospective cohort study was conducted in a sample of 3,565 women with live births. Participants were classified by mode of conception as follows: fertile women who conceived naturally (fertile women; *n* = 2,857), women with infertility who conceived naturally (sub-fertile women; *n* = 483), and women with infertility who conceived through ART (women with infertility; *n* = 310). The infant-feeding patterns were assessed with four-time points before two months postpartum. Binary and multinomial logistic regression and causal mediation analyses were performed.

**Results:**

The rates of breastfeeding initiation and discontinuation across modes of conception were similar. However, infertile and sub-fertile women had 37% (95% CI 1.02, 1.83) and 56% (95% CI 1.06, 2.27) increased risks of introducing formula before the first week postpartum, respectively, and 35% (95% CI 1.01, 1.82) and 52% (95% CI 1.04, 2.24) higher risks of exclusive breastfeeding for less than one week, respectively, compared to fertile women. The relationships were mainly mediated through multiple gestation and admission to neonatal/pediatric intensive care units (NICU/PICU; proportions of mediation were over 50%). The effects of mode of conception on breastfeeding outcomes became not significant in cases of singleton birth.

**Conclusions:**

Sub-fertile women and women with infertility intended to breastfeed but experienced higher perinatal risks in the early postpartum period. Multiple gestation and admission to NICU/PICU forced them to introduce formula earlier than preferred, thus leading to a shorter duration of exclusive breastfeeding. Single embryo transfer policy and breastfeeding support in NICU/PICU could help those women achieve positive early breastfeeding outcomes.

## Introduction

The rates of childbirth resulting from Assisted Reproductive Technology (ART) are increasing worldwide [1]; however, pregnancies resulting from ART are at higher risks for adverse birth outcomes (e.g., preterm birth and low birthweight) [2–4]. Continuous breastfeeding may aid growth and development in children because human breastmilk contains unique nutrients that promote neuronal, intestinal, and immunological development [5, 6].

Nonetheless, studies demonstrate that women who conceived through ART cease breastfeeding earlier or breastfeed for shorter durations than those who conceived naturally; however, contrary findings indicate no such association [7–12]. This inconsistency may owe to study limitations, including lack of appropriate comparison groups, insufficient sample sizes, and failure to control the effects of characteristics of the setting where women go for prenatal visits or to give birth. Additionally, a study showed that the first two months postpartum is the most influential period for professional support to prolong breastfeeding [13]; still, most studies assessed outcomes only at the one-time point during the first two months. Hence, they could not determine the timing of the emergence of breastfeeding differences.

Additionally, if an association exists, a systematic exploration of the potential pathways between the modes of conception and early breastfeeding outcomes has yet to be conducted. Understanding such possible pathways may help provide evidence-based strategies to improve breastfeeding. Previous studies show that women who conceived through ART are at a higher risk for pregnancy/delivery complications [2–4]. Such experiences and treatments could delay breast fullness owing to elevated stress hormones [14]. Additionally, pregnancies resulting from ART are at higher risk for low birth weight, preterm birth, and admission to neonatal/pediatric intensive care units (NICU/PICU) [2, 4, 15]. Babies with poor health status often experience difficulty in latching and result in ineffective suckling [16]. This can negatively influence mothers’ perception of the adequacy of their milk production [17]. Furthermore, considering that multiple births are more prevalent among mothers who conceived through ART, some may experience increased care burden and difficulty establishing sufficient milk supply for the infants [18]. Hence, in this study, the potential mediators—pregnancy and delivery complications—were considered in the pathway of maternal health factors; the potential mediators—multiple gestation, low birth weight, preterm birth, and admission to NICUs/PICUs—were considered in the pathway of infant health factors.

**Fig 1:**
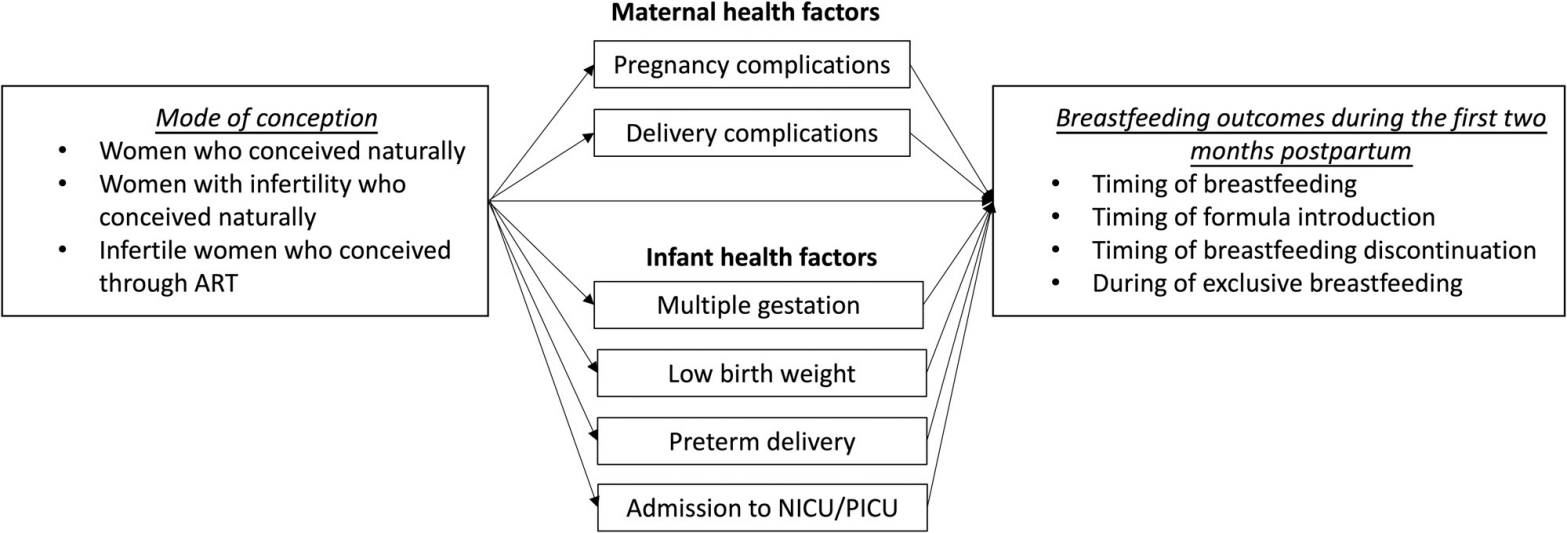
Hypothesised model.

Therefore, this study aimed to examine the effect of mode of conception on breastfeeding outcomes during the first two months postpartum and identify and quantify how much of the effects are mediated through maternal and infant health factors (Fig 1).

## Materials and methods

### Study design and setting of the study

A retrospective cohort study was conducted. The source population was women who had live births in a medical center in northern Taiwan from January 2014 to December 2018. The reason for choosing this period was that the national surveys indicated similar breastfeeding rates at the first and second month postpartum during this period [19]; this choice could thereby reduce differences caused by time trends or other unmeasured factors. The participating medical center was in northern Taiwan. It had an infertility clinic and a department of Obstetrics and received the certificate for implementing a Baby-Friendly Hospital Initiative (BFHI) for more than two decades, ensuring that all postpartum women receive similar prenatal care and breastfeeding counseling. Generally, the BFHI complied with the “Ten Steps to Successful Breastfeeding” framework, developed by the WHO and UNICEF [20]. We assessed the source population’s exposure status from January 1999 (i.e., the current electronic medical records system was initiated), and participants were followed up until the second month postpartum.

The data were compiled from medical records, birth registrations, medical materials and examinations systems, and breastfeeding follow-up records. Breastfeeding follow-up records were kept by trained nurses who identified breastfeeding outcomes through face-to-face interviews during hospital stays and telephone interviews after discharge; these were conducted at one week, one month, and two months postpartum. The breastfeeding follow-ups had been incorporated into nursing routines since the implementation of the BFHI.

An experienced obstetrician and an epidemiologist with experience in study design and analysis using medical records and birth certification databases scrutinized and assessed whether the measurements of each variable appeared to be a good measure to achieve face validity of the data. This study was approved by the institutional ethics review board from the study hospital (NO. 20190402R) and performed following the Declaration of Helsinki and the relevant regulations. Because this was a retrospective analysis of administrative data using anonymized patient identity, waiving informed consent was granted.

### Participants

There were 8,830 women with live births, 3,356 of whom were excluded. The exclusion criteria were: age under 20 years (*n* = 43), fewer than five prenatal visits (*n* = 863), infants with congenital defects (*n* = 48), unmarried status (*n* = 265), insurance status below low income (*n* = 1), and probable fertility treatment outside the hospital (*n* = 2,091), which were identified through those who delivered by a certain obstetrician who admitted most patients from an outside infertility clinic. Mothers with fewer than five prenatal visits were excluded to ensure that the participants received similar breastfeeding consultation from conception to breastfeeding; unmarried mothers were excluded because the Assisted Reproduction Act in Taiwan allows ART only for married couples. Women who conceived through intrauterine insemination (*n* = 45) were excluded because this procedure is not defined as ART [21].

**Fig 2:**
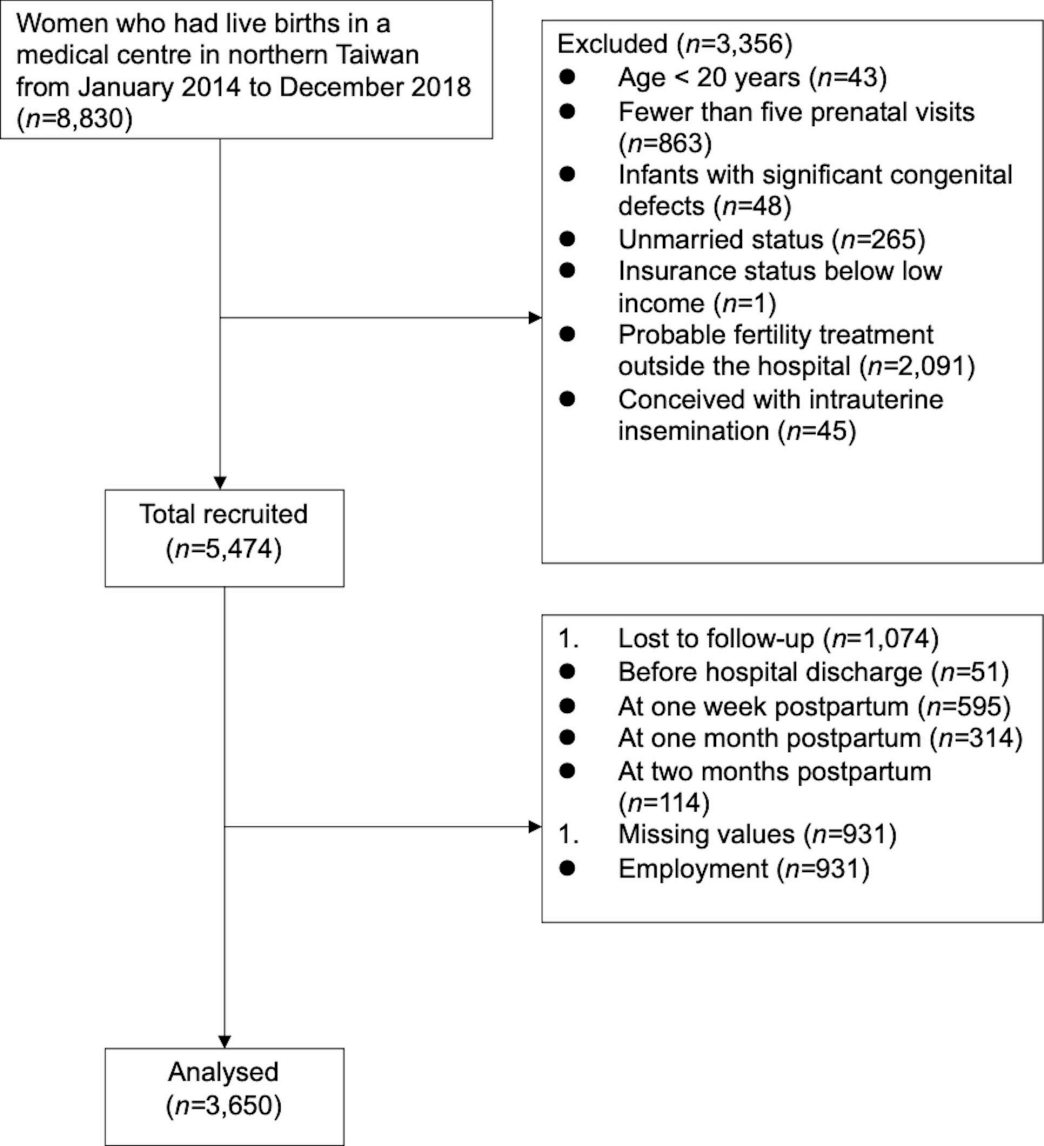
Flowchart of participants.

After the four follow-ups, it was noticed that 19.62% (n = 1,074) of the participants were unable to be followed up because they refused to be interviewed during hospital stays or did not answer phone calls after hospital discharge; still, 80.38% (*n* = 4,400) of our sample completed the four follow-ups. Considering the missing values on employment (n = 931), a final sample of 3,650 women with complete information was obtained (Fig 2).

### Exposure

By mode of conception, participants were categorized into three groups: fertile women who conceived naturally; women with infertility who conceived naturally (i.e., sub-fertile women); and women with infertility who conceived through ART (i.e., women with infertility). As medical records did not clearly indicate mothers’ mode of conception, the three groups were defined as follows. Infertility was defined as the failure to achieve a clinical pregnancy after more than 12 months of regular unprotected sexual intercourse and being identified with an infertility diagnosis (i.e., International Classification of Diseases (ICD)-9 628.0 to 628.9 or ICD-10 N97.0 to N97.9) through medical records. Women with no infertility diagnosis and no history of ART were defined as fertile women. To identify whether the pregnancy was conceived through ART, ART conception was defined as cases in which the offspring’s birth date must be within 266 days after the date of implantation. The following calculation determined the value of 266 days: 280 days subtracting by 14 days–the duration between last menstrual period and the retrievals, subtracting by 5 days–accounts for 3–5 days in embryo culture [22], and adding 3 days of errors [23]. Women diagnosed with infertility but had no history of ART or for whom the offspring’s birth date was not within the 266 days after the implantation date (whichever met) were classified as sub-fertile women.

### Outcomes

#### Timing of breastfeeding initiation, formula introduction, and breastfeeding discontinuation

The infant feeding patterns included exclusive and mixed breastfeeding and formula feeding; these were assessed with four-time points–before the first week postpartum, the first week, the first month, and the second month postpartum. The timing of breastfeeding initiation was divided into before or after the first week postpartum because most women initiated breastfeeding before the first week postpartum. The timing of formula introduction was defined as the first-time introduction of formula during the follow-up period. It was classified into five groups: less than a week, one week, one month, two months after birth, and no formula introduction within two months postpartum. Breastfeeding discontinuation was defined as the discontinuation of breastfeeding during the follow-up period. It was classified into five groups: less than a week, one week, one month, two months after birth, and no breastfeeding discontinuation within two months postpartum.

#### Duration of exclusive breastfeeding

Infant breastfeeding practice intervals were converted into days and summed to compute the duration of exclusive breastfeeding. This allowed us to identify changes in infant feeding practices (e.g., from formula feeding to exclusive breastfeeding). Exclusive breastfeeding indicated that the infant received only breast milk, without any other liquids/solids. As only a tiny proportion of participants (6.0%) exclusively breastfed for one week to less than one month, the duration from one week to less than one month and from one month to less than two months were merged into one category. Therefore, the duration of exclusive breastfeeding was categorized into four groups: less than one week, one week, one week to less than two months, and two months.

### Confounders

Based on previous studies and the assumption of a possible underlying mechanism, we tested for the minimally sufficient adjustment set of confounders using a causal directed acyclic graph created with DAGitty (see S1 Fig); this served to avoid the risk of over-adjustment or improper adjustment for mediators [24, 25]. The final models included the following confounders: maternal age, maternal occupational status, abortion history, parity, and pre-existing diseases.

### Potential mediators

As shown in Fig 1, two mediating pathways were examined—maternal health and infant health. Maternal health factors included pregnancy complications (i.e., gestational diabetes mellitus, oligohydramnios, gestational hypertension, and pre-eclampsia) and delivery complications (i.e., premature rupture of membranes, abruptio placentae, placenta previa, postpartum hemorrhage, prolonged labor, obstructed labor, malpresentation, and prolapsed umbilical cord), data for which were obtained from birth registrations. Infant health factors included multiple gestation, low birth weight (<2,500 g), preterm birth (<37 weeks), and admission to NICU/PICU.

### Statistical analysis

All statistical analyses were performed using STATA version 15.0 and RStudio version 1.4.1106 [26, 27]. Maternal and infant characteristics were described using counts and percentages by mode of conception and compared between groups using linear regression for continuous variables and binary logistic regression for dichotomous variables. To estimate odds ratios (ORs), relative risk ratios (RRRs), and 95% confidence intervals (CIs) for the association between the mode of conception and breastfeeding outcomes during the first two months postpartum adjusting for the confounders, binary and multinomial logistic regressions were used. The reference group consisted of fertile women. All *P*-values were two-tailed with a significance level of 5%.

To identify the mediating pathways through maternal and child health factors, a causal mediation analysis was conducted. The criteria for a mediator were examined by binary or multinomial logistic regression as follows: (1) a model for the mediator, conditional on the mode of conception and confounders; and (2) a model for breastfeeding outcomes, conditional on the mode of conception, the mediator, and the same confounders [28]. To test a *priori* the hypothesized mechanisms, only mediators of interest that met the criteria were entered into the causal mediation analysis. A causal mediation analysis was performed using the method developed by Shih et al. that can estimate the mediation effect under the setting of a dichotomous mediator and outcome without the rare outcome assumption [29]. The mediation analysis was briefly summarized as follows. First, we specified a joint model for the outcome and mediator with two logistic regressions.

$$logit[P(Y=1|E,M,C)]=\beta_{0}+\beta_{e}E+\beta_{m}M+\beta_{em}E\times M+\beta_{c}^{T}c$$ (1)

$$logit[P(M=1|E,C)]=\alpha_{0}+\alpha_{q}E+\alpha_{x}^{T}c$$ (2)

where *Y*, *E*, *M*, and *C* were breastfeeding outcomes, mode of conception, mediators, and confounders, respectively. Second, we used the following formula to obtain natural direct (NDE) and indirect (NIE) effects:

$$NDE=\sum_{m\in \{0,1\}}\left\{\tau (m,e_{a}=1,e_{b}=0,C)-\tau (m,e_{a}=0,e_{b}=0,C)\right\}$$

$$NIE=\sum_{m\in \{0,1\}}\left\{\tau (m,e_{a}=1,e_{b}=1,C)-\tau (m,e_{a}=1,e_{b}=0,C)\right\},$$

where $\tau (m,e_{a},e_{b},c)=\left(\frac{{exp(\beta }_{0}+\beta_{e}e_{a}{+\beta }_{m}m+\beta_{em}e_{a}m+\beta_{c}^{T}c)}{1+{exp(\beta }_{0}+\beta_{e}e_{a}{+\beta }_{m}m+\beta_{em}e_{a}m+\beta_{c}^{T}c)}\right)\left(\frac{\exp\left\{(\alpha_{0}+\alpha_{q}e_{b}+\alpha_{x}^{T}c)m\right\}}{1+\exp\left(\alpha_{0}+\alpha_{q}e_{b}+\alpha_{x}^{T}c\right)}\right)$, and the parameters {*α*_0_, *α*_*q*_, ***α***_*X*_} and {*β*_0_, *β*_*e*_, *β*_*m*_, *β*_*em*_, ***β***_*c*_} were estimated from the models (1) and (2), respectively.

The detailed methodology of the mediation analyses can be found elsewhere [29]. The total effects of the mode of conception on breastfeeding outcomes were divided into (1) effects exerted through mediators (natural indirect effect, NIE); and (2) effects not exerted through mediators (natural direct effect, NDE). CIs of direct and indirect effects were estimated from 1,000 bootstrapping replicates. The proportion mediated was calculated as [log (OR^NIE^)/ log (OR^NDE^×OR^NIE^)] ×100% to interpret the proportion of the total effect explained by the mediation.

#### Sensitivity analyses

To deal with missing data on employment (*n* = 931, 17.01%) and breastfeeding practices (*n* = 1,074, 19.62%), repeated analyses using the multivariate imputation by chained equations (MICE) method were performed [30]. Twenty imputations were used to reduce the sampling error, and Rubin’s combination rules were used to consolidate the obtained estimates [31].

Moreover, the primary analyses limited to singleton pregnancy were performed to further examine the role of plurality, as multiple gestation may have been the upstream factor for delivery complications, low birth weight, preterm delivery, and admission to NICU/PICU. An E-value analysis was performed to estimate how large at least an unmeasured confounder must be to render the observed association a null estimate [32].

## Results

### Maternal and infant characteristics by mode of conception

Among the 3,650 participants, 2,857 (78.3%) were fertile, 483 (13.2%) were sub-fertile, and 310 (8.5%) were women with infertility who conceived through ART. The maternal and infant characteristics by mode of conception are presented in Table 1. Both sub-fertile and women with infertility were more likely to be older, primiparous, and to have a history of abortion and pre-existing diseases than fertile women. Women with infertility were less likely to be employed compared with fertile women. Regarding maternal and infant health conditions, sub-fertile women and women with infertility were more likely to have the delivery complication, multiple gestation, low birth weight, preterm birth, and admission to NICU/PICU than fertile women. However, sub-fertile women and women with infertility had a similar rate of pregnancy complications compared with fertile women.

**Table 1:** Maternal and infant characteristics by mode of conception (N = 3,650).

Characteristics	Fertile women (*n* = 2,857)	Sub-fertile women (*n* = 483)	*P*-value	Women with infertility (*n =* 310)	*P*-value
Maternal age, mean ± *SD*	32.76 ± 0.09	35.15 ± 0.18	< .001	36.52 ± 0.19	< .001
Employment status, n (%)					
Unemployed	829 (29.02)	145 (30.02)	0.65	120 (38.71)	< .001
Part-time job/full-time job	2028 (70.98)	338 (69.98)		190 (61.29)	
Parity, n (%)			< .001		< .001
1	1704 (59.64)	330 (68.32)		227 (73.23)	
≥ 2	1153 (40.36)	153 (31.68)		83 (26.77)	
Abortion history, n (%)			0.006		0.04
No	1843(64.51)	280 (57.97)		182 (58.71)	
Yes	1014 (35.49)	203 (42.03)		128 (41.29)	
Pre-existing diseases, n (%)			< .001		< .001
No	2563 (89.71)	378 (78.26)		248 (80.00)	
Yes	294 (10.29)	105(21.74)		62 (20.00)	
Pregnancy complications, n (%)			0.07		0.11
No	2753 (96.36)	457 (94.62)		293 (94.52)	
Yes	104 (3.64)	26 (5.38)		17 (5.48)	
Delivery complications, n (%)			< .001		< .001
No	2065 (72.28)	308 (63.77)		168 (54.19)	
Yes	792 (27.72)	175 (36.23)		142 (45.81)	
Multiple gestation, n (%)			< .001		< .001
No	2828 (98.98)	453 (93.79)		255 (82.26)	
Yes	29 (1.02)	30 (6.21)		55 (17.74)	
Low birth weight, n (%)			0.01		< .001
No	2702 (94.57)	443 (91.72)		258 (83.23)	
Yes	155 (5.43)	40 (8.28)		52 (16.77)	
Preterm birth, n (%)			< .001		< .001
No	2720 (95.24)	437 (90.48)		258 (83.23)	
Yes	136 (4.76)	46 (9.52)		52 (16.77)	
Admission to NICU/PICU, n (%)			< .001		< .001
No	2481 (86.84)	375 (77.64)		233 (75.16)	
Yes	376 (13.16)	108 (22.36)		77 (24.84)	

*Note*. *P*-values were computed using linear regression for continuous response variables and binary logistic regression for dichotomous response variables. The reference group consisted of fertile women. *NICU/PICU*, neonatal intensive care unit/pediatric intensive care unit; *SD*, standard deviation.

### Association between mode of conception and early breastfeeding outcomes

Both sub-fertile women and women with infertility were more likely to introduce formula before the first week postpartum than fertile women by 37% (adjusted RRR = 1.37, 95% CI = 1.02, 1.83) and 56% (adjusted RRR = 1.56, 95% CI = 1.06, 2.27), respectively. Additionally, during the first two months postpartum, both sub-fertile women and women with infertility had a higher risk of exclusive breastfeeding for less than seven days compared with fertile women by 35% (adjusted RRR = 1.35, 95% CI = 1.01, 1.82) and 52% (adjusted RRR = 1.52, 95% CI = 1.04, 2.24), respectively. Neither the timing of breastfeeding initiation nor breastfeeding discontinuation was associated with the mode of conception (Table 2).

**Table 2:** Association between mode of conception and early breastfeeding outcomes (N = 3,650).

Early breastfeeding outcomes	*n*	(%)	Sub-fertile women			Women with infertility		
			Adjusted RRR	(95% CI)	*P*-value	Adjusted RRR	(95% CI)	*P*-value
Timing of breastfeeding initiation								
≤1 week	3583	(98.16)	1.00	(Reference)		1.00	(Reference)	
>1 week	67	(1.84)	0.56 ^a^	(0.23, 1.33)	0.10	0.92 ^a^	(0.40, 2.14)	0.47
Timing of introduction of formula								
No formula introduction	714	(19.56)	1.00	(Reference)		1.00	(Reference)	
<1 week postpartum	1991	(54.55)	1.37	(1.02, 1.83)	0.04	1.56	(1.06, 2.27)	0.02
1^st^ week postpartum	312	(8.56)	1.21	(0.79, 1.87)	0.38	0.86	(0.47, 1.57)	0.62
1^st^ month postpartum	571	(15.64)	0.80	(0.54, 1.18)	0.27	0.72	(0.43, 1.22)	0.22
2^nd^ month postpartum	62	(1.69)	1.44	(0.68, 3.07)	0.34	0.24	(0.03, 1.83)	0.17
Timing of discontinuing any breastfeeding								
No breastfeeding discontinuation	2825	(77.40)	1.00	(Reference)		1.00	(Reference)	
<1 week postpartum	67	(1.84)	0.57	(0.24, 1.37)	0.21	0.95	(0.41, 2.21)	0.91
1^st^ week postpartum	58	(1.59)	1.83	(0.96, 3.51)	0.07	0.93	(0.35, 2.49)	0.89
1^st^ month postpartum	238	(7.75)	1.14	(0.77, 1.67)	0.52	1.28	(0.79, 2.08)	0.31
2^nd^ month postpartum	417	(11.42)	1.04	(0.76, 1.43)	0.80	1.16	(0.78, 1.71)	0.45
Duration of exclusive breastfeeding								
2 months	683	(18.71)	1.00	(Reference)		1.00	(Reference)	
<1 week	1694	(46.41)	1.35	(1.01, 1.82)	0.04	1.52	(1.04, 2.24)	0.03
1 week	557	(15.26)	0.79	(0.54, 1.18)	0.22	0.67	(0.40, 1.15)	0.13
>1 week to <2 months	716	(19.62)	1.35	(0.96, 1.89)	0.09	1.26	(0.81, 1.96)	0.32

*Note*. Adjusted relative risk ratios were estimated using multinomial logistic regression, except for timing of breastfeeding initiation presenting as odds ratios using binary logistic regression, after adjusting for maternal age, maternal occupational status, abortion history, parity, and pre-existing diseases. Reference group for mode of conception was fertile women.

^a^Adjusted odds ratios; *RRR*, relative risk ratios; *ref*., reference group; *CI*, confidence interval.

### Causal mediation analyses

All mediators of interest in the hypothesized model met the mediator criteria (see S1 and S2 Tables) and were included in the causal mediation analysis, except for pregnancy complications. Table 3 presented each mediator of the association with introducing formula before the first week postpartum and exclusive breastfeeding for less than one week, respectively, for the mode of conception.

**Table 3:** Causal mediation analysis: Effect of mode of conception on breastfeeding outcomes through mediators.

Mediators	Sub-fertile women →introduction of formula at ≤1^st^ week postpartum					Women with infertility→ introduction of formula at ≤1^st^ week postpartum					Sub-fertile women →exclusive breastfeeding for less than one week					Women with infertility → exclusive breastfeeding for less than one week				
	OR^NDE^	*P*-value	OR^NIE^	*P*-value	PM%	OR^NDE^	*P*-value	OR^NIE^	*P*-value	PM%	OR^NDE^	*P*-value	OR^NIE^	*P*-value	PM%	OR^NDE^	*P*-value	OR^NIE^	*P*-value	PM%
Delivery complications	1.38	0.04	1.01	0.80	NA	1.44	0.04	1.07	0.01	16.15	1.31	0.04	1.02	0.37	NA	1.38	0.04	1.07	0.01	17.34
Multiple gestation	1.30	0.09	1.06	0.003	18.78	1.23	0.29	1.27	< .001	53.30	1.23	0.13	1.07	0.004	23.77	1.18	0.42	1.23	0.001	55.82
Preterm birth	1.34	0.05	1.03	0.04	7.73	1.39	0.13	1.09	0.001	20.69	1.28	0.11	1.03	0.02	9.54	1.38	0.22	1.07	0.005	16.92
Low birth weight	1.36	0.05	1.03	0.04	7.43	1.40	0.13	1.10	0.003	21.59	1.29	0.07	1.03	0.04	8.94	1.35	0.19	1.10	0.004	23.12
Admission to NICU/PICU	1.17	0.28	1.18	0.002	50.81	1.29	0.22	1.16	0.02	36.29	1.15	0.25	1.16	< .001	52.49	1.20	0.34	1.22	0.005	51.81

*Note*. Mediators of interest that did not meet the mediator criteria are not shown. All models were adjusted for confounders—maternal age, maternal occupational status, abortion history, parity, and pre-existing diseases. *OR*^*NDE*^, odds ratios of natural direct effect; *OR*^*NIE*^, odds ratios of natural indirect effect; *PM*, proportion mediated; *NICU/PICU*, neonatal intensive care unit/pediatric intensive care unit; *NA*, not applicable due to the nonsignificant indirect effect. Reference group for mode of conception was fertile women. Reference group for timing of introduction of formula was no formula introduction within two months postpartum. Reference group for duration of exclusive breastfeeding was two months.

The association between sub-fertile women and the introduction of formula before one week postpartum was mediated by infant health factors including multiple gestations (proportion mediated, PM, 18.78%), preterm (PM, 7.72%), low birth weight (PM, 7.43%), and admission to NICU/PICU (PM, 50.81%). The mediation proportion of the total effect of sub-fertile women on less than a week of exclusive breastfeeding was similar to that for the effect of on the introduction of formula before one week postpartum (PM, 23.77% for multiple gestation, 9.54% for preterm, 8.94% for low birth weight, and 52.49% for admission to NICU/PICU).

The association between women with infertility and the introduction of formula before one week postpartum could be mediated by delivery complications, of which the proportion mediated accounted for 16.15%. Additionally, the association was mediated mainly by infant health factors including multiple gestation (53.30%), low birth weight (21.59%), preterm birth (20.69%), and admission to NICU/PICU (36.29%). The mediation proportion of the association between women with infertility and exclusive breastfeeding for less than one week was 17.34% through delivery complications, 55.82% through multiple gestations, 23.12% through low birth weight, 16.92% through preterm birth, and 51.81% through admission to NICU/PICU.

### Sensitivity analysis

When repeating the analyses using the multivariate imputation method to deal with the missing data, the results did not change the study’s findings with complete data (see S3 Table). Among women with a singleton gestation, the association between mode of conception and the timing of formula introduction and exclusive breastfeeding duration, respectively, were no longer significant (Table 4). Namely, multiple gestation may be an upstream mediator in the association between mode of conception and poor early breastfeeding outcomes. Sensitivity analyses showed that the required unmeasured confounder to explain away the associations with introducing formula before the first week postpartum were 2.08-fold for sub-fertile women and 2.49-fold for women with infertility. Regarding the associations with less than the one-week duration of exclusive breastfeeding, the required unmeasured confounder to explain away the associations was 2.04-fold for sub-fertile women and 2.41-fold for women with infertility.

**Table 4:** Association between mode of conception and early breastfeeding outcomes in women with singleton gestations (N = 3,536).

Early breast-feeding outcomes	Sub-fertile women			Women with infertility		
	Adjusted RRR	(95% CI)	*P*-value	Adjusted RRR	(95% CI)	*P*-value
Timing of introduction of formula						
No formula introduction)	1.00	(Reference)		1.00	(Reference)	
< 1 week postpartum	1.29	(0.96, 1.74)	0.09	1.24	(0.84, 1.85)	0.28
1^st^ week postpartum	1.24	(0.80, 1.90)	0.33	0.83	(0.45, 1.55)	0.57
1^st^ month postpartum	0.82	(0.55, 1.21)	0.32	0.72	(0.42, 1.22)	0.22
2^nd^ month postpartum	1.47	(0.69, 3.14)	0.32	0.25	(0.03, 1.90)	0.18
Duration of exclusive breastfeeding						
2 months	1.00	(Reference)		1.00	(Reference)	
< 1 week	1.22	(0.90, 1.65)	0.20	1.18	(0.79, 1.76)	0.43
1 week	0.80	(0.54, 1.18)	0.26	0.66	(0.38, 1.13)	0.13
> 1 week to < 2 months	1.36	(0.97, 1.91)	0.08	1.12	(0.71, 1.78)	0.62

*Note*. *CI*, confidence interval; *RRR*, relative risk ratios; Adjusted relative risk ratios were adjusted for maternal age, maternal occupational status, abortion history, parity, and pre-existing diseases. Reference group for mode of conception was fertile women.

## Discussion

Our findings suggested that during the first two months postpartum, both sub-fertile women and women with infertility had a shorter duration of exclusive breastfeeding (i.e., less than one week) and an earlier introduction of formula (i.e., before the first week postpartum) but did not cease breastfeeding early. These findings echoed those of a previous study, which suggested that women who undergo ART exclusively breastfeed for a shorter duration compared with fertile women [7]. We further added a piece of evidence that they were more likely to be introducing formula earlier rather than completely stopping breastfeeding. An earlier introduction of formula could lead to insufficient milk production, which increases in severity over time, and thereby results in a shorter exclusive breastfeeding duration.

Breastfeeding intention is a strong predictor of breastfeeding initiation [33]. Hence, our finding of similar rates of breastfeeding initiation and discontinuation across modes of conception indicated that sub-fertile women and women with infertility intended to breastfeed, but experienced difficulties in the early postpartum period that forced them to introduce formula earlier than preferred, leading to a shorter duration of exclusive breastfeeding.

This study further found that the differences in the timing of formula introduction between the mode of conception occurred before the first week postpartum; in other words, the earliest difference occurred while mothers and infants were still in the hospital. This provided a targeted period and suggested that healthcare professionals pay more attention to sub-fertile women and women with infertility who conceived through ART on their breastfeeding difficulties during hospital stays.

The present study also provided the mechanisms for the association between mode of conception and early formula introduction and shorter duration of exclusive breastfeeding. This study demonstrated that multiple gestation is an essential and upstream mediator in the association between women with infertility conceiving through ART and early breastfeeding outcomes. Ovulation induction therapies and transferring multiple embryos during ART are well-documented risk factors for multiple gestations [34, 35]. Multiple gestation is associated with higher rates of delivery complications, low birth weight, and admission to NICU, often due to prematurity [36–38]. This may explain why the effect of women with infertility on poor early breastfeeding outcomes is strongly mediated by multiple gestation. Single embryo transfer is the most effective strategy to reduce the multiple gestation rates related to ART. However, the uptake and acceptance of single embryo transfer still varied across countries [39, 40]. In Taiwan, the Assisted Reproduction Act allows for the implantation of fewer than five embryos and encourages transferring two or one embryos for women aged less than 35 years [41]. Even so, transferring two or more embryos accounted for about 80% of all embryo transfer cycles [42]. This study provided evidence of the additional benefit of a single embryo transfer policy that could help narrow the disparity in breastfeeding outcomes between women with infertility and fertile women. Therefore, introducing a single embryo transfer policy is suggested to improve perinatal outcomes, together with the following breastfeeding outcomes. Single embryo transfer should be further advocated in Taiwan. Giving this information to those women who plan to breastfeed is also suggested to consider when choosing the number of transferred embryos.

Apart from the mediation effects caused by multiple gestation, the effects of mode of conception on the introduction of formula and duration of exclusive breastfeeding can be mediated largely through admission to NICU/PICU. This highlights the importance of practices in the NICU/PICU; therefore, targeting both sub-fertile women and women with infertility and developing a lactation support program in the NICU/PICU is important. This remark becomes especially relevant if we consider the increased number of births resulting from ART [43]. Moreover, not all NICU admissions are necessary. For example, two studies remarked on the occurrence of unnecessary NICU admissions in recent years [44, 45]. Thus, to reduce unnecessary NICU admissions, the need to scrutinize and loosen the NICU admission criteria was also suggested; doing so may thereby decrease adverse breastfeeding events resulting from an unnecessary NICU admission.

### Strengths of the study

We investigated the relationship between mode of conception—as categorized into three groups—and early breastfeeding outcomes during the first two months postpartum, a topic that has been sub-optimally approached in the literature. Previous studies have not examined whether sub-fertile women and women with infertility displayed different effects regarding early breastfeeding outcomes. Moreover, our assessment took place across multiple time points, allowing for the investigation of the emergence of breastfeeding differences. Additionally, we identified the maternal and infant health pathways using formal causal mediation analysis to provide evidence-based strategies to improve breastfeeding.

### Limitations

First, our results were based on data from a single hospital; thus, the generalizability of our findings is limited. Second, given that this was a retrospective cohort study, data collection was restricted to past medical records; thus, our study lacked information on staffs’ and family members’ recommendations and behaviors regarding birth complications, all of which may influence breastfeeding behaviors. We also could not obtain information about the specific type of ART and the use of drugs. Still, there is no evidence supporting that different types of ART or drugs might lead to a differential effect on breastfeeding behavior. Third, although we controlled or adjusted several confounders, residual confounding may still exist. However, the sensitivity analysis using E-value methodology indicated that the observed association could only be explained by an unmeasured confounder that associated with both mode of conception and early breastfeeding outcomes by a risk ratio of more than 2.04. Given that this risk ratio is much greater than any observed for known risk factors examined in the current study, it is implausible that an unmeasured confounder exists that can overcome the effect. Finally, our causal mediation models assumed parallel mediation because a sequential causal mediation software for multiple categorical mediators, which could be used to identify numerous specific pathway effects when a correlation exists, was unavailable [46]. However, we detected the most upstream mediator–multiple gestation–and important setting–NICU/PICU–to intervene.

## Conclusion

Sub-fertile women and women with infertility who conceived through ART were more likely to introduce formula before the first week postpartum and have an exclusive breastfeeding duration of less than one week during the first two months, than fertile women. The timings of breastfeeding initiation and discontinuation were similar among mode of conception. In the relationship between modes of conception and the two significant early breastfeeding outcomes, multiple gestation may be an upstream mediator, and admission to NICU/PICU serves as the other important mediator. Targeting and designing programs to reduce multiple gestation and admission to NICU/PICU were suggested to diminish the differences in breastfeeding among the mode of conception. Single embryo transfer policy and breastfeeding support in NICU/PICU could help sub-fertile women and women with infertility achieve positive early breastfeeding outcomes.

## Supporting information

S1 Fig Directed acyclic graph. Created with DAGitty (www.dagitty.net). NICU/PICU, neonatal intensive care unit/pediatric intensive care unit; LBW, low birth weight. The variables with pink color indicate confounders, which require controlling or adjusting. The blue variables with green arrows indicate mediators, which cannot be controlled or adjusted for. The minimal sufficient adjustment sets for estimating the total effect of mode of conception on early breastfeeding outcomes include maternal age, maternal occupational status, abortion history, parity, marital status, socioeconomic status, and pre-existing diseases.
(TIF)

S1 Table Coefficient estimations in models for mediators and mode of conception as independent variables. (DOCX)

S2 Table Coefficient estimations in models for breast-feeding outcomes and mediators as independent variables. (DOCX)

S3 Table Association between mode of conception and early breast-feeding outcomes after using MICE method. (DOCX)
