# Supplementary figures and images for: Why does mode of conception affect early breastfeeding outcomes? A retrospective cohort study
Source: PLoS One. 2022 Mar 18;17(3):e0265776. doi: 10.1371/journal.pone.0265776 (PMC8932581; doi:10.1371/journal.pone.0265776)

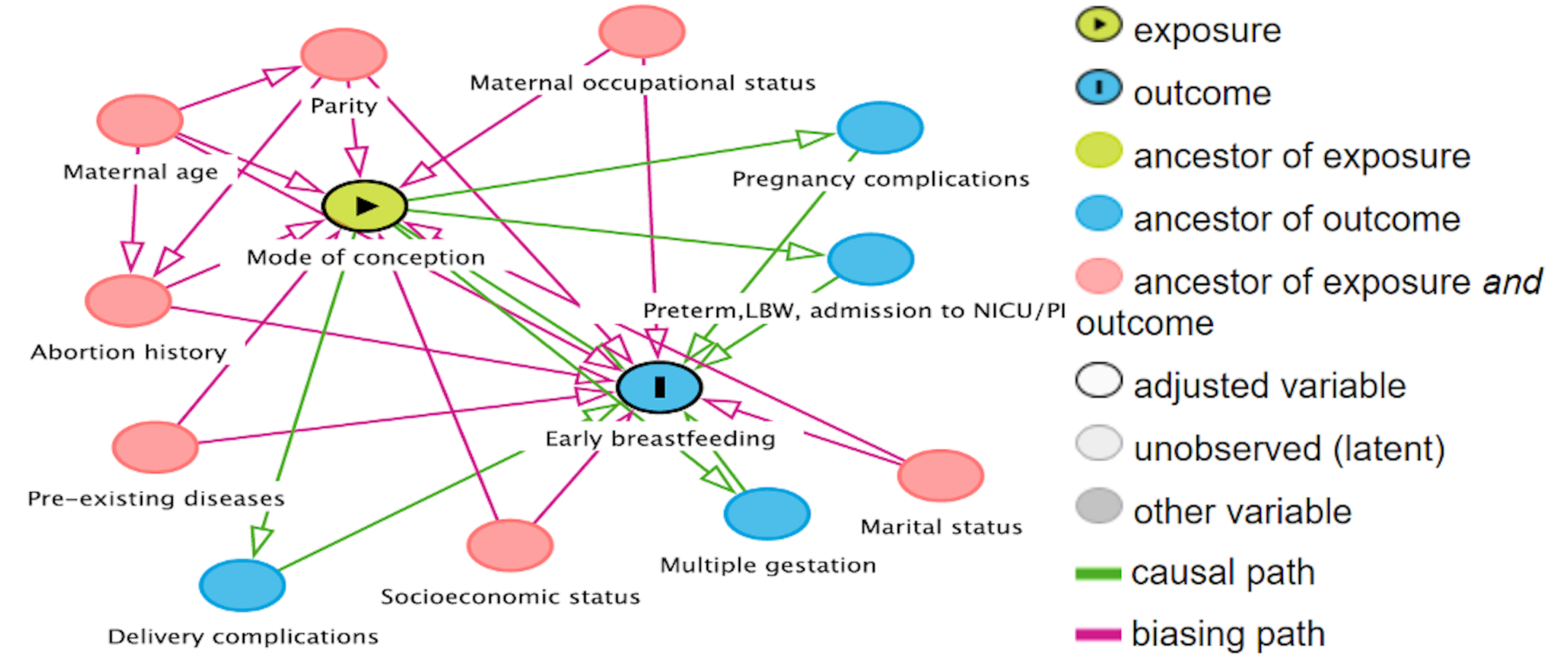

Supplement: S1 Fig — Created with DAGitty (www.dagitty.net). NICU/PICU, neonatal intensive care unit/pediatric intensive care unit; LBW, low birth weight. The variables with pink color indicate confounders, which require controlling or adjusting. The blue variables with green arrows indicate mediators, which cannot be controlled or adjusted for. The minimal sufficient adjustment sets for estimating the total effect of mode of conception on early breastfeeding outcomes include maternal age, maternal occupational status, abortion history, parity, marital status, socioeconomic status, and pre-existing diseases. (TIF) [file pone.0265776.s001.tif]
