# Supplementary material for: Why does mode of conception affect early breastfeeding outcomes? A retrospective cohort study
Source: PLoS One. 2022 Mar 18;17(3):e0265776. doi: 10.1371/journal.pone.0265776 (PMC8932581; doi:10.1371/journal.pone.0265776)
Supplement: S1 Table — (DOCX) [file pone.0265776.s002.docx]

**S1 Table.** Coefficient estimations in models for mediators and mode of conception as independent variables

| **Potential mediators** | **Mode of conception** **(reference= Fertile women)** | | | |
| --- | --- | --- | --- | --- |
|  | **Sub-fertile women** | ***P*-value** | **Women with infertility** | ***P*-value** |
| Pregnancy complications |  |  |  |  |
| No | (Reference) |  | (Reference) |  |
| Yes | 0.24 | 0.30 | 0.18 | 0.53 |
| Delivery complications |  |  |  |  |
| No | (Reference) |  | (Reference) |  |
| Yes | 0.18 | 0.04 | 0.48 | <.001 |
| Multiple gestation |  |  |  |  |
| No | (Reference) |  | (Reference) |  |
| Yes | 1.8 | <.001 | 2.91 | <.001 |
| Low birth weight |  |  |  |  |
| ≥ 2,500 g | (Reference) |  | (Reference) |  |
| < 2,500 g | 0.37 | 0.04 | 1.12 | <.001 |
| Preterm |  |  |  |  |
| ≥ 37 weeks | (Reference) |  | (Reference) |  |
| < 37 weeks | 0.70 | <.001 | 1.29 | <.001 |
| Admission to NICU/PICU |  |  |  |  |
| No | (Reference) |  | (Reference) |  |
| Yes | 0.57 | <.001 | 0.66 | <.001 |

*Note.* *ART*, assisted reproductive technology; *NICU/PICU*, neonatal intensive care unit/pediatric intensive care unit. Coefficient estimates were generated by multinomial logistic regression and adjusted for maternal age, maternal occupational status, abortion history, parity, and pre-existing diseases.
