# Supplementary material for: Why does mode of conception affect early breastfeeding outcomes? A retrospective cohort study
Source: PLoS One. 2022 Mar 18;17(3):e0265776. doi: 10.1371/journal.pone.0265776 (PMC8932581; doi:10.1371/journal.pone.0265776)
Supplement: S2 Table — (DOCX) [file pone.0265776.s003.docx]

**S2 Table.** Coefficient estimations in models for breast-feeding outcomes and mediators as independent variables

| **Potential mediators** | **Timing of introduction of formula with continued breast feeding (reference = no introduction of formula)** | | | | | | | |  | **Duration of exclusive breast feeding**  **(reference =2 months)** | | | | | |
| --- | --- | --- | --- | --- | --- | --- | --- | --- | --- | --- | --- | --- | --- | --- | --- |
|  | **< 1 week** | ***P*-value** | **1 week** | ***P*-value** | **1 month** | ***P*-value** | **2 months** | ***P*-value** |  | **< 1 week** | ***P*-value** | **1 week** | ***P*-value** | **>1 week to <2 months** | ***P*-value** |
| Pregnancy complications |  |  |  |  |  |  |  |  |  |  |  |  |  |  |  |
| No | (Reference) |  | (Reference) |  | (Reference) |  | (Reference) |  |  | (Reference) |  | (Reference) |  | (Reference) |  |
| Yes | 0.94 | 0.002 | 1.24 | 0.001 | 0.14 | 0.74 | -0.12 | 0.91 |  | 1.09 | <.001 | 0.13 | 0.76 | 0.59 | 0.09 |
| Delivery complications |  |  |  |  |  |  |  |  |  |  |  |  |  |  |  |
| No | (Reference) |  | (Reference) |  | (Reference) |  | (Reference) |  |  | (Reference) |  | (Reference) |  | (Reference) |  |
| Yes | 0.81 | <.001 | -0.61 | 0.002 | -0.46 | 0.003 | -0.06 | 0.87 |  | 0.67 | <.001 | -0.46 | 0.002 | 0.53 | <.001 |
| Multiple gestation |  |  |  |  |  |  |  |  |  |  |  |  |  |  |  |
| No | (Reference) |  | (Reference) |  | (Reference) |  | (Reference) |  |  | (Reference) |  | (Reference) |  | (Reference) |  |
| Yes | 2.32 | <.001 | -0.50 | 0.67 | -0.33 | 0.72 | -12.51 | 0.99 |  | 2.31 | <.001 | -1.04 | 0.37 | 1.29 | 0.05 |
| Low birth weight |  |  |  |  |  |  |  |  |  |  |  |  |  |  |  |
| ≥ 2,500 g | (Reference) |  | (Reference) |  | (Reference) |  | (Reference) |  |  | (Reference) |  | (Reference) |  | (Reference) |  |
| < 2,500 g | 1.19 | <.001 | 0.59 | 0.09 | -0.02 | 0.95 | -13.58 | 0.98 |  | 1.12 | <.001 | 0.18 | 0.58 | 0.89 | 0.001 |
| Preterm |  |  |  |  |  |  |  |  |  |  |  |  |  |  |  |
| ≥ 37 weeks | (Reference) |  | (Reference) |  | (Reference) |  | (Reference) |  |  | (Reference) |  | (Reference) |  | (Reference) |  |
| < 37 weeks | 1.13 | <.001 | -0.01 | 0.98 | 0.02 | 0.95 | -0.61 | 0.55 |  | 1.02 | <.001 | 0.004 | 0.99 | 0.89 | 0.001 |
| Admission to NICU/PICU |  |  |  |  |  |  |  |  |  |  |  |  |  |  |  |
| No | (Reference) |  | (Reference) |  | (Reference) |  | (Reference) |  |  | (Reference) |  | (Reference) |  | (Reference) |  |
| Yes | 4.13 | <.001 | 2.37 | <.001 | -1.18 | 0.29 | 1.05 | 0.35 |  | 3.87 | <.001 | -0.53 | 0.55 | 3.89 | <.001 |

*Note.* *ref.*, reference group; *NICU/PICU*, neonatal intensive care unit/pediatric intensive care unit. Coefficient estimates were generated by binary or multinomial logistic regression and adjusted for mode of conception, maternal age, maternal occupational status, abortion history, parity, and pre-existing diseases.
