# Supplementary material for: Why does mode of conception affect early breastfeeding outcomes? A retrospective cohort study
Source: PLoS One. 2022 Mar 18;17(3):e0265776. doi: 10.1371/journal.pone.0265776 (PMC8932581; doi:10.1371/journal.pone.0265776)
Supplement: S3 Table — (DOCX) [file pone.0265776.s004.docx]

**S3 Table.** Association between mode of conception and early breast-feeding outcomes after using MICE method(N=5,474)

| **Early breastfeeding outcomes** | **Sub-fertile women** | | |  | **Women with infertility** | | |
| --- | --- | --- | --- | --- | --- | --- | --- |
|  | **Adjusted RRR** | **(95% CI)** | ***P*-value** |  | **Adjusted RRR** | **(95% CI)** | ***P*-value** |
| Timing of breastfeeding initiation |  |  |  |  |  |  |  |
| ≤1 week | 1.00 | (Reference) |  |  | 1.00 | (Reference) |  |
| >1 week | 0.85 ^a^ | (0.46, 1.60) | 0.62 |  | 1.20 ^a^ | (0.60, 2.43) | 0.61 |
| Timing of introduction of formula |  |  |  |  |  |  |  |
| No formula introduction | 1.00 | (Reference) |  |  | 1.00 | (Reference) |  |
| <1 week postpartum | 1.34 | (1.03, 1.73) | 0.03 |  | 1.60 | (1.11, 2.31) | 0.01 |
| 1^st^ week postpartum | 1.26 | (0.85, 1.86) | 0.26 |  | 1.01 | (0.57, 1.79) | 0.97 |
| 1^st^ month postpartum | 0.87 | (0.61, 1.24) | 0.43 |  | 0.91 | (0.56, 1.47) | 0.70 |
| 2^nd^ month postpartum | 1.44 | (0.73, 2.84) | 0.29 |  | 0.30 | (0.04, 2.31) | 0.25 |
| Timing of discontinuing any breastfeeding |  |  |  |  |  |  |  |
| No breastfeeding discontinuation | 1.00 | (Reference) |  |  | 1.00 | (Reference) |  |
| <1 week postpartum | 0.85 | (0.45, 1.59) | 0.61 |  | 1.25 | (0.62, 2.52) | 0.54 |
| 1^st^ week postpartum | 1.78 | (0.98, 3.23) | 0.06 |  | 0.94 | (0.36, 2.45) | 0.90 |
| 1^st^ month postpartum | 0.94 | (0.68, 1.31) | 0.73 |  | 1.10 | (0.73, 1.68) | 0.64 |
| 2^nd^ month postpartum | 0.99 | (0.76, 1.29) | 0.94 |  | 1.16 | (0.83, 1.63) | 0.38 |
| Duration of exclusive breastfeeding |  |  |  |  |  |  |  |
| 2 months | 1.00 | (Reference) |  |  | 1.00 | (Reference) |  |
| <1 week | 1.31 | (1.01, 1.70) | 0.04 |  | 1.59 | (1.10, 2.31) | 0.01 |
| 1 week | 0.87 | (0.64, 1.24) | 0.48 |  | 0.76 | (0.46, 1.24) | 0.27 |
| >1 week to <2 months | 1.28 | (0.95, 1.71) | 0.10 |  | 1.31 | (0.86, 1.98) | 0.21 |

*Note*. Adjusted relative risk ratios were estimated using multinomial logistic regression, except for timing of breastfeeding initiation presenting as odds ratios using binary logistic regression, after adjusting for maternal age, maternal occupational status, abortion history, parity, and pre-existing diseases.

^a^ Adjusted odds ratios; MICE, multivariate imputation by chained equations; *RRR*, relative risk ratios; *ref.*, reference group; *CI*, confidence interval.
